# Patient-Reported Outcomes Before and After Hallux Valgus Surgery: 2-Year Results From a National Registry Study

**DOI:** 10.1177/24730114251351634

**Published:** 2025-07-24

**Authors:** Eva Tengman, Cyrus Brodén, Ann-Charlott Söderpalm, Maria C. Cöster

**Affiliations:** 1 Department of Community Medicine and Rehabilitation, Umeå University, Sweden; 2 Department of Surgical Sciences, Orthopaedics and Handsurgery, Uppsala University, Sweden; 3 Department of Orthopedics and Clinical Sciences in Gothenburg, Sahlgrenska Academy, Sweden

**Keywords:** hallux valgus, patient-reported outcome measure, National Quality Register, EQ-5D, SEFAS, Swefoot registry

## Abstract

**Background:**

Hallux valgus (HV) is a common condition associated with pain, functional limitations, and reduced health-related quality of life. Population-level data on 2-year results of surgical treatment remain limited. The aim of this study was to evaluate patient-reported outcome measures (PROMs) before surgery, and 1- and 2-year follow-up in patients registered in the Swedish quality registry for foot and ankle surgery (“Swefoot”) who underwent surgery for HV. Further, the relation between PROMs and HV severity, BMI, and smoking habits was explored.

**Methods:**

Seven hundred sixty-five feet from 742 patients (median age 58, 87% women) who underwent primary surgery for HV were included. Data on demographics, comorbidities, HV severity, surgical procedures, and PROMs were extracted. PROMs included the Self-Reported Foot and Ankle score (SEFAS), EuroQol 5-dimensional, 3-level (EQ-5D), and satisfaction regarding surgery, appearance, shoe wear, and pain.

**Results:**

At both 1- and 2-year follow-ups, approximately 80% of responding patients reported satisfaction with the surgical outcome (80.4% and 81.0%, respectively). Patients reported significant improvements in SEFAS scores: mean increase of 10 points (95% CI 9.1-10.4) at 1 year and 11 points (95% CI 9.8-11.2) at 2 years compared to baseline. Health-related quality of life also improved significantly; EQ-5D VAS was improved with 7 (95% CI 5-8) after 1 year and EQ-5D index was improved with 0.19 (95% CI 0.17-0.21). The EQ-5D VAS and index did not change significantly between the 1- and 2-year follow-ups.

Significant improvements were also reported in satisfaction with appearance, shoe wear, and pain. Regardless of HV grade, the patients had similar improvement in SEFAS. Overweight/obese patients experienced similar improvements as underweight/normal BMI ones. Smokers showed significantly less improvement in SEFAS.

**Conclusion:**

This population-based study using the Swefoot registry data demonstrates that patients surgically treated for HV reported significant improvements in PROMs from before surgery to 1 and 2 years after surgery.

**Level of Evidence:**

Level II, prognostic study.

## Introduction

Hallux valgus (HV) is a common forefoot deformity, with a global prevalence of 19%; it is more common in women (24%) than men (11%), and among individuals older than 60 years (23%).^5^ Individuals with HV report pain, dissatisfaction regarding appearance, difficulty wearing footwear, and, especially in the elderly, difficulty balancing and risk of falling.^24-26,31^ In recent years, patient-reported outcome measures (PROMs) have been increasingly used in clinical studies of the foot and ankle to assess health-related quality of life (HrQoL) before and after surgery. Several studies have reported that HV is associated with impaired HrQoL and that the deterioration is related to the severity of the HV deformity.^1,16,35^ HV is classified by Mann and Coughlin into 3 grades based on the intermetatarsal angle (IMA) and hallux valgus angle (HVA), as mild (IMA < 11 degrees, HVA < 20 degrees), moderate (IMA 11-16 degrees, HVA 20-40 degrees), and severe (IMA > 16 degrees, HVA > 40 degrees).^13^ Surgical treatment for HV deformity is superior to nonsurgical treatment,^15^ and numerous surgical procedures have been described. These can be categorized into osteotomies, soft-tissue procedures, and fusions,^35^ and are used either individually or in combination with one another. HrQoL improves after HV surgery as measured by both generic and region-specific PROMs.^17,28^ In HV research, the American Orthopaedic Foot & Ankle Society (AOFAS) ankle-hindfoot score has been most frequently used despite the fact that this score is both clinician- and patient-based, which means it is not a true PROM. In recent years, PROMs with good quality, that is, with good measurement properties, have been developed and used. The Manchester Oxford Foot Questionnaire (MOXFQ) and the Self-reported Outcome Score (SEFAS) are suitable PROMs to assess HV surgery.^34,36^

Swefoot registry, the Swedish Quality Registry for Foot and Ankle Surgery, collects data on surgically treated foot and ankle conditions, including HV. The registry includes data provided by patients and surgeons, collected from both public and private health care units that perform foot and ankle surgery in Sweden.^9^ In a recently published register study using Swefoot registry data, HrQoL was found to be affected by all grades of HV, and that patients with comorbidities such as obesity and rheumatoid arthritis are even more affected.^35^ However, further knowledge is needed regarding the outcomes of surgery in large studies based on national registries. Additionally, it is of interest to investigate whether foot- and ankle-specific results vary based on the severity of HV and modifiable factors such as body mass index (BMI) and smoking habits.

Therefore, the aim of this study was to evaluate patient-reported outcomes before surgery and 1 and 2 years after surgery in patients included in Swefoot registry who were surgically treated for HV between December 2016 and May 2019. A further aim was to explore whether the HV severity, BMI, and smoking habits affect patient-reported foot- and ankle-related outcomes.

## Methods

### Study Design and Participants

**Figure 1.**
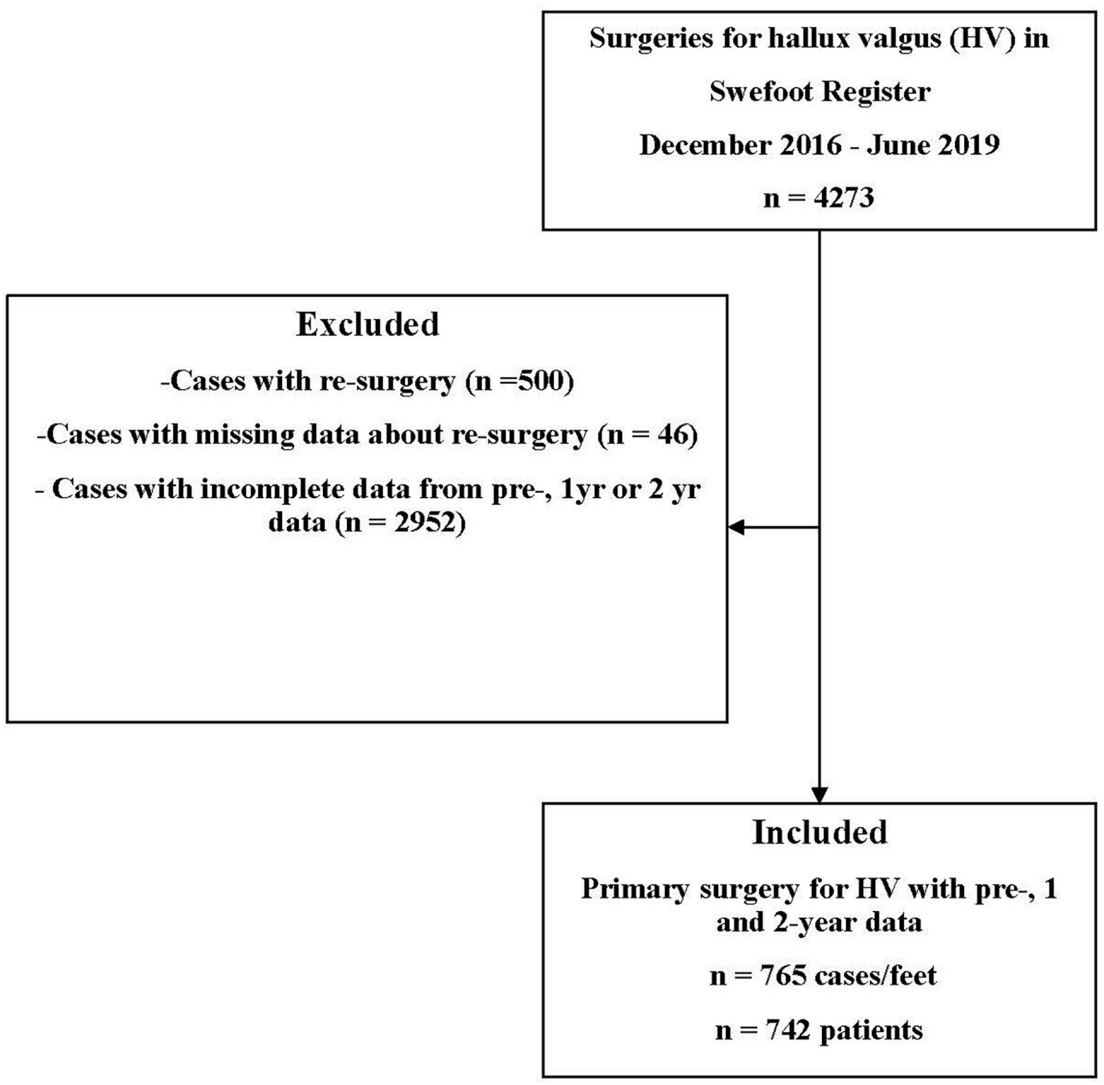
Flowchart showing the selection process for the study population.

In this registry-based study, data were retrieved in May 2021. The registry was established in 2014, with 2-year follow-ups beginning in December 2016. Data included 4273 feet that had been surgically treated for HV between December 2016 and May 2019. Only patients who had undergone primary surgery and for whom patient-reported data from presurgery and the 1- and 2-year follow-ups were included. The present study included a total of 765 feet in 742 patients. The exclusion criteria are shown in Figure 1. We extracted data on age, sex, BMI, smoking habits, comorbidities, grading of HV, surgical procedures, and patient-reported data, including the PROMs SEFAS, EQ-5D, and self-reported satisfaction.

### Swefoot Registry (the Swedish Quality Registry for Foot and Ankle Surgery)

Both surgeons and patients report data to Swefoot registry (introduced in 2014).^9^ Baseline preoperative information such as sex, age, anthropometric measurements (height, weight, BMI), comorbidities, and smoking habits are registered. At the time of surgery, the surgeon provides data about the diagnosis, previous surgery, grading of deformity, concomitant arthrosis, other deformities, surgical methods, and postoperative regimen. The patient completes 2 patient-reported outcome measures (PROMs): the Self-reported Foot and Ankle Score (SEFAS), and the EuroQol 5-dimensional, 3-level (EQ-5D) instrument before surgery and 1 and 2 years postsurgery. Additionally, the patient responds to questions concerning appearance, shoe wear, and forefoot pain. At the 1- and 2-year follow-ups, they also respond to a specific question pertaining to their satisfaction with the outcome.

### Patient-Reported Outcome Measures and Patient-Reported Satisfaction

SEFAS is a foot- and ankle-specific PROM that covers constructs including pain, functional limitation, and HRQoL. The psychometric properties of SEFAS have been thoroughly evaluated for conditions affecting both the forefoot, including hallux valgus, and hindfoot.^6,8,11^ The SEFAS contains 12 questions, with 5 answer options (0-4); a summary score of 0 indicates severe disability, and 48 indicates normal function. The minimally important change (MIC) values reflect the smallest measured change in score that patients perceive as being important, and population-based normative values are available for SEFAS.^10,12^ The MIC value for forefoot disorders is 5 score points. The population-based normative SEFAS value for women aged 50-59 years is 42 (SD 9) and for men 46 (SD 5).^12^ SEFAS is one of the recommended PROMs for evaluating hallux valgus.^34^

The EQ-5D is a validated generic PROM for assessing HrQoL.^4^ It is validated for patients with foot and ankle disorders and considers mobility, self-care, usual activities, pain/discomfort, and anxiety/depression. Each dimension has 3 levels: no problems, some problems, and extreme problems. A summary index is calculated after adjustment for cultural differences, and the UK EQ-5D Tariff is applied to compute an index score ranging from zero to 1.0, with the latter denoting full health.^38^ The EQ-5D-VAS is a health evaluation that uses a visual analog scale (VAS) from 0 to 100.

The questions regarding satisfaction with surgery results, appearance, and shoe wear have 5 response options: very satisfied, satisfied, somewhat satisfied (categorized as satisfied), neither satisfied nor dissatisfied, and dissatisfied (categorized as not satisfied). For the question concerning forefoot pain, the response options are none, mild, (categorized as no), moderate, strong, and severe (categorized as yes).

### Statistical Analysis

All statistical analyses were performed using the Statistical Package for the Social Sciences, version 28 (IBM SPSS, Armonk, NY). Continuous data were tested for normality using the Shapiro-Wilk test; Levene test was used to test homogeneity. Data are presented with mean, 95% CI, median, and range. Categorical data are presented as numbers with percentages. Repeated measures analysis of variance with Bonferroni post hoc tests were used to compare SEFAS and EQ-5D VAS and scores presurgery and 1 and 2 years after surgery. To analyze the effects of HV grade, BMI, and smoking habits on SEFAS outcomes, repeated analyses of variance with Bonferroni post hoc tests were used, including HV grade, BMI, and smoking habits as factors. For categorical variables, χ^2^ and Fisher exact tests were used to analyze the differences between presurgery and 1-year follow-up, and between presurgery and 2-year follow-up. Because of multiple statistical comparisons, the significance level was set at *P* < .005.

### Ethics

The study protocol received approval from the Ethical Review Board in Sweden (reference number 2019-02733), and adhered to the Helsinki Protocol. Data registration and study implementation were conducted confidentially following patient consent, and in accordance with Swedish and EU data-protection regulations. Data accessibility was possible through application to the register. The study received support from “Stiftelsen för Skobranschens utvecklingsfond.” The national registry, Swefoot, is funded by the Swedish Association of Local Authorities and Regions. The funders did not influence the study design, data collection, analysis, interpretation, manuscript composition, or any other study aspect. The authors report no conflicts of interest.

## Results

### Patient Characteristics

The study included 765 feet (742 patients) that had been surgically treated for HV and for whom there was data for presurgery and the 1- and 2-year follow-ups. The patients had a median age of 58 years (range 16-90). Most patients were women (87%). Mean BMI was 25.9, with 51.7% having a BMI higher than 25. Sixteen percent of patients had comorbidities: either rheumatic or diabetic diseases. A moderate grade of HV was most common (59.7%), followed by severe (24.2%) and mild (16.1%) (Table 1). Twenty-three patients had surgery on both their right and left feet, with a median time between the surgeries of 4 months (range 0-19 months).

**Table 1.** Participant characteristics. Background data are presented using median (range), mean (SD), or number (percentages) across all participants, and within women or men specifically.

	All	Women	Men
Participants n (%)	765	663 (87)	102 (13)
Age (y), median (range)	58 (16-90)	57 (17-90)	61 (16-84)
Height (cm), mean (SD)	167.8 (7.7)	166.0 (6.1)	179,7 (6.3)
Weight (kg), mean (SD)	73.1 (13.4)	71.1 (12.0)	86.6 (14.4)
BMI, mean (SD)	25.9 (4.0)	25.8 (4.1)	26.7 (3.7)
Smoking, n (%)			
Nonsmoker	681 (92)	594 (92.4)	87 (89.7)
Smokers	18 (2.4)	13 (2.0)	5 (5.2)
Quit smoking	41 (5.5)	36 (5.6)	5 (5.2)
Prerheumatic, n (%)	89 (12.1)	79 (12.4)	10 (10.1)
Prediabetic, n (%)	32 (4.2)	27 (4.1)	5 (5.0)
HV grade, n (%)			
Mild	122 (16.1)	109 (16.6)	13 (13.0)
Moderate	451 (59.7)	399 (60.8)	52 (52.0)
Severe	183 (24.2)	148 (22.5)	35 (35.0)
Type of surgery, n (%)			
Distal osteotomy	572 (74.8)	508 (76.6)	64 (62.7)
Shaft osteotomy	135 (17.6)	108 (16.3)	27 (26.4)
Other	58 (7.6)	47 (7.1)	11 (10.8)

### Patient-Reported Outcome Measures and Patient-Reported Satisfaction

The patients reported significant improvements in terms of SEFAS. A significant improvement was seen between presurgery and the 1-year follow-up, with mean 10 (95% CI 9.1-10.6) score points. There was an even larger improvement between presurgery and the 2-year follow-up, with 11 (95% CI 9.8-11.2) score points (Table 2).

**Table 2.** 

	Presurgery	1-y Follow-up	2-y Follow-up	Mean Difference (95% CI)	*F*	SE	Significance^a^ (*P*)
SEFAS (n = 660)					708		**<.001**
Mean	29.6	39.4	40.1	Preop–1 y: 9.8 (8.9 to 10.6)		0.34	**<.001**
95% CI	29.1 to 30.2	38.8 to 40.0	39.5 to 40.7	Preop–2 y: 10.5 (9.6 to 11.3)		0.35	**<.001**
				1-2 y: 0.7 (0.2 to 1.3)		0.23	**.004**
EQ-5D VAS (n = 666)					60.6		**<.001**
Mean	75	81	80	Preop–1 y: 6.7 (5.0 to 8.4)		0.72	**<.001**
95% CI	73 to 76	80 to 83	79 to 82	Preop–2 y: 5.8 (4.1 to 7.6)		0.73	**<.001**
				1-2 y: −0.9 (−2.1 to 0.3)		0.51	.251
EQ-5D index (n = 627)					246		**.001**
Mean	0.65	0.84	0.84	Preop–1 y: 0.19 (0.16 to 0.21)		0.011	**.001**
95% CI	0.63 to 0.67	0.83 to 0.85	0.82 to 0.85	Preop–2 y: 0.18 (0.16 to 0.21)		0.011	**.001**
				1-2 y: −0.004 (−0.21 to 0.01)		0.007	>.999

Abbreviations: SEFAS, Self-reported Foot and Ankle Score; EQ-5D, EuroQol 5-dimensional; VAS, visual analog scale.

a Significance presents *P* values from the repeated analysis of variance Bonferroni post hoc tests between time points. Significance level was set to *P* ≤ .005.

The patients also reported significant improvements in generic health (HrQoL), as measured by the EQ-5D VAS and index. Compared with the baseline, EQ-5D VAS scores improved by 7 (95% CI 5-8) 1 year after surgery, and by 6 (95% CI 4-8) 2 years after surgery. Likewise, for the EQ-5D index, significant improvements were seen: 0.19 (95% CI 0.16-0.21) after 1 year, and 0.18 (95% CI 0.16-0.21) after 2 years. The EQ-5D VAS and index did not change significantly between the 1- and 2-year follow-ups (Table 2).

Roughly 80% of the patients expressed satisfaction with the surgery outcome at the 1-year (80.4%) and 2-year (81.0%) follow-ups. Significant improvements were seen for self-reported satisfaction regarding appearance, shoe wear, and pain in the forefoot (Table 3).

**Table 3.** Patient-reported satisfaction with surgery outcome at one- and two-year follow-ups, and satisfaction regarding appearance, shoe wear, self-reported strength, and pain in the forefoot pre-surgery and at one-and two-year follow-ups. The options for general satisfaction regarding surgery, appearance, shoe wear, and strength were very satisfied, satisfied, somewhat satisfied (categorized as satisfied = yes), neither satisfied nor dissatisfied, and dissatisfied (categorized as not satisfied = no). The options for pain in the forefoot were none, mild, (categorized as no), moderate, strong, and severe (categorized as yes). Chi-squared and Fisher’s exact tests were used to analyze the differences between pre-surgery and the one-year follow-up, and between pre-surgery and the two-year follow-up.

	Presurgery	1-y Follow-up	2-y Follow-up	Significance^a^
Satisfaction with surgery outcome, n (%)				
Yes	–	615 (80.4)	618 (81.0)	–
No	–	150 (19.6)	145 (19.0)	–
Satisfaction with appearance, n (%)				
Yes	142 (18.6)	610 (80.1)	595 (78.2)	Preop–1 y: ***p* < .001**
No	620 (81.4)	152 (19.9)	166 (21.8)	Preop–2 y: *p* = .013
Satisfaction with shoe wear, n (%)				
Yes	233 (30.7)	633 (83.5)	618 (81.6)	Preop–1 y: ***p* < .001**
No	527 (69.3)	125 (16.5)	139 (18.4)	Preop–2 y: *p* = .015
Pain in the forefoot, n (%)				
Yes	587 (76.9)	241 (32.4)	229 (32.4)	Preop–1 y: *p* = .012
No	176 (23.1)	503 (67.6)	478 (67.6)	Preop–2 y: ***p* = .002**

a Boldface indicates significance (*P* ≤ .005).

### SEFAS in Relation to Grade of Hallux Valgus, BMI, and Smoking Habits

**Figure 2.**
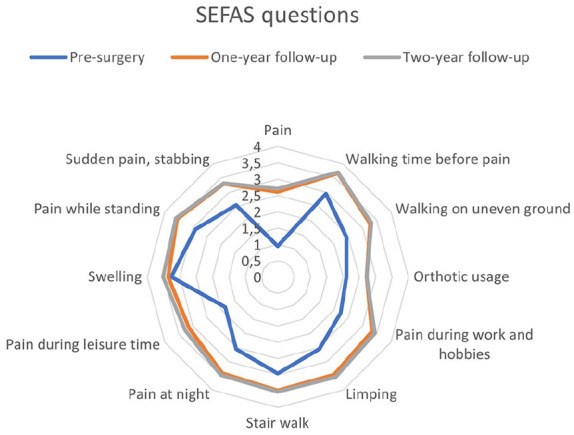
The diagram presents the mean points for the 12 specific SEFAS questions. Each question has 5 answer options (0-4), where a higher score indicates better function and/or less pain. The 3 lines represent the 3 time points: the blue line shows the presurgery score, the red line shows the 1-year follow-up score, and the gray line shows the 2-year follow-up score. SEFAS, Self-reported Foot and Ankle Score.

Certain SEFAS subquestions were more affected and had greater improvement following surgery where the largest improvement was observed in the questions relating to pain and its effects on leisure activities time (Figure 2).

**Figure 3.**
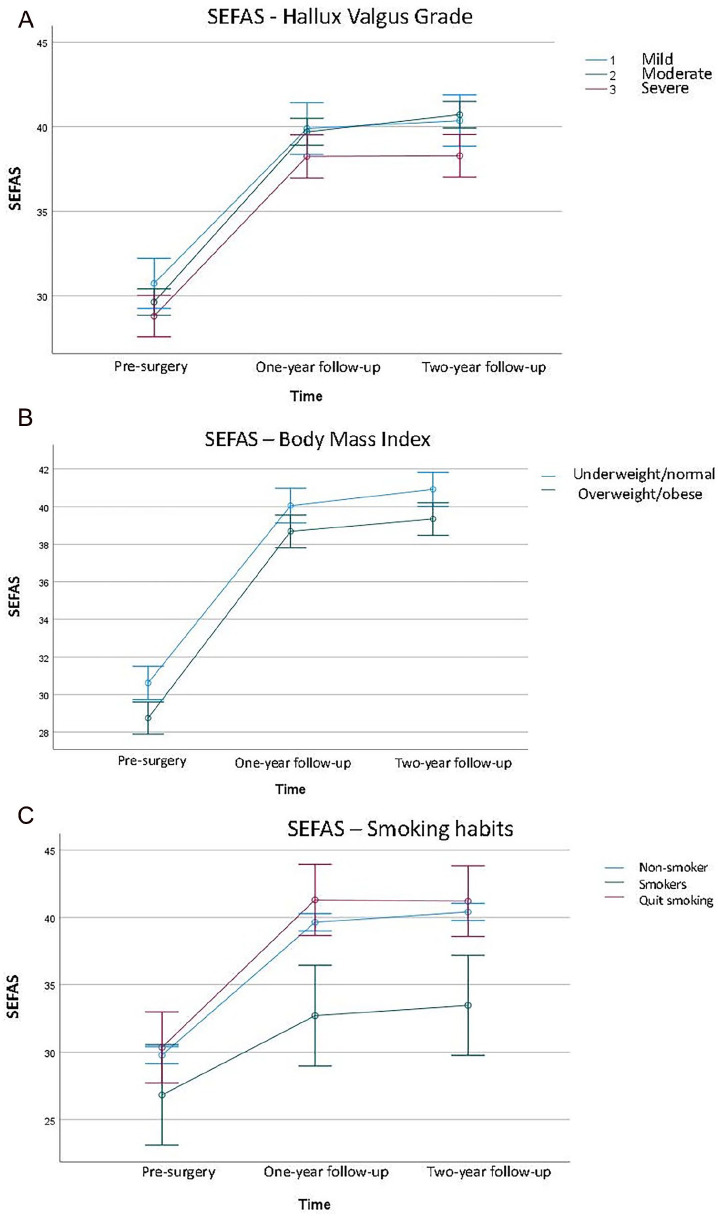
(A-C) SEFAS analyzed with repeated measures analysis of variance and the figures are presenting SEFAS mean score and 95% CI at the 3 time points: presurgery and 1- and 2-year follow-ups. (A) SEFAS outcome in relation to the presurgery grades of hallux valgus (mild, moderate, severe). (B) SEFAS outcome in relation to BMI (underweight/normal and overweight/obese). (C) SEFAS outcome in relation to smoking habits (nonsmoker, smoker, and quit smoking before surgery). SEFAS, Self-reported Foot and Ankle Score.

Regardless of presurgery HV grade (mild, moderate, or severe), all patients had similar significant improvements in the SEFAS score (*P* < .001) (Figure 3A, Table 4). Persons with overweight or obesity generally had lower SEFAS scores (mean 1.6 score points *P* = .002); however, the improvement was similar (Figure 3B, Table 4). Smokers had significantly lower SEFAS scores (mean 5.6, 6.6 score points, *P* = .001) and lower improvements (mean 5.9, 6.7 score points) at the 1- and 2-year follow-ups compared with nonsmokers and individuals who had quit smoking before the surgery (Figure 3C, Table 4).

**Table 4.** Pre-surgery and one- and two-year follow-up data for SEFAS in relation to HV grade, BMI, and smoking habits. Data are presented in mean (95 % CI lower-upper bound). Repeated analyses of variance with Bonferroni post hoc tests were used, including HV grade, BMI, and smoking habits as factors.^a^

				Between-Subject	Within-Subject	
	Presurgery	1-y Follow-up	2-y Follow-up	Significance: Factor^b^	Significance: Time^c^	Significance: Interaction^d^
SEFAS (n = 656)				*F* = 3.9 *P* = .021	*F* = 491, ***P* < .001**	*F* = 1.5, *P* = .199
Mild	30.7 (29.3 to 32.2)	39.9 (38.4 to 41.4)	40.4 (38.9 to 41.9)		Preop–1 y: ***P* < .001**	
Moderate	29.6 (28.9 to 30.4)	39.7 (28.9 to 40.5)	40.7 (39.9 to 41.5)		Preop–2 y: ***P* < .001**	
Severe	28.8 (27.6 to 30.0)	38.2 (37.0 to 39.5)	38.3 (37.0 to 39.5)		1-2 y: *P* = .159	
SEFAS (n = 618)				*F* = 9.5, ***P* = .002**	*F* = 65, ***P* < .001**	*F* = 0.31, *P* = .735
Underweight/normal	30.6 (29.7 to 31.5)	40.1 (39.1 to 41.0)	40.9 (40.0 to 41.8)		Preop–1 y: ***P* < .001**	
Overweight/obese	28.7 (27.9 to 29.6)	38.7 (37.8 to 39.6)	39.3 (38.5 to 40.2)		Preop–2 y: ***P* < .001**	
					1-2 y: ***P* = .003**	
SEFAS (n = 638)				*F* = 7.0, ***P* < .001**	*F* = 88, ***P* < .001**	*F* = 1.56, *P* = .181
Nonsmoker	29.8 (29.1 to 30.4)	39.6 (39.0 to 40.3)	40.4 (39.8 to 41.0)	Nonmoker vs Smoker: ***P* < .001**	Preop–1 y: ***P* < .001**	
Smokers	26.8 (23.1 to 30.6)	32.7 (29.0 to 36.4)	33.5 (29.8 to 37.2)	Nonsmoker vs Quit smoking: *P* = 1.00	Preop–2 y: ***P* < .001**	
Quit smoking	30.4 (27.7 to 33.0)	41.3 (38.7 to 43.9)	41.2 (38.6 to 43.8)	Quit smoking vs Smokers: ***P* < .001**	1-2 y: *P* = 1.00	

Abbreviation: SEFAS, Self-reported Foot and Ankle Score.

a Significance presents *P* values from the repeated measures analysis of variance Bonferroni post hoc analyses. Significance level was set to *P* ≤ 0.005. Boldface indicates significance.

b Factor refers to HV grade, BMI cat, smoking habits.

c Time refers to presurgery, 1 year follow-up, 2 years follow-up.

d Interaction refers to interaction between time and factor.

## Discussion

In this registry-based study of 765 feet (742 patients), patient-reported outcomes were evaluated presurgery and at 1 and 2 years postoperatively. HV surgeries were found to improve self-reported outcomes (SEFAS and EQ-5D) significantly and durably, with clinically meaningful gains maintained at 2-year follow-up. Significant improvements were also observed in patient satisfaction with appearance, shoe wear, and forefoot pain relief, with approximately 80% of responding patients expressing satisfaction with surgery at both follow-up time points. Smoking was found to have a negative effect on patient-reported results after surgery.

### Comparisons With Other Studies

Our study found significant improvements in the foot- and ankle-specific PROM (SEFAS) at 1- and 2-year follow-ups, and they were similar regardless of the severity of the HV before surgery. The MIC value for SEFAS is 5 points in forefoot patients^10^; that is, the improvements were also clinically relevant. The results of this large register-based study support previous results found in a cohort of 50 patients, with all but the most severe of HV deformities, with near-identical improvements as in the present study 1 and 2 years after surgery, respectively.^28^ The SEFAS scores at both 1- and 2-year follow-up after surgery are comparable to normative data,^12^ suggesting that HV patients almost completely recover after surgery. EQ-5D data are also comparable with data from the above-presented cohort of 50 patients. The improvement from before to after surgery was 0.22 compared with 0.19 in our study.^28^ Other recently published studies have found improvements in EQ-5D index between 0.11 and 0.26 after 1 year.^16,23^

A systematic review conducted in 2020 that included 12 studies with 1313 patients explored the effects of HV surgery on HrQoL, finding that few studies describe the results of HV surgery in terms of HrQoL and concluding that further research is required. The 12 included studies showed that HV surgery resulted in decreased body pain, improved physical function, and improved social QoL domain, but no change in the mental QoL domain.^17^ It has been shown that HV patients with poorer preoperative mental health have worse postoperative improvement in QoL^41^; these relationships should be evaluated in future studies, as well as using a national cohort.

The EQ-5D dimensions can be analyzed separately^7^; however, in the present study, no in-depth analyses of these dimensions were conducted. We did explore the different SEFAS questions and found that the pain dimension was an important part of the improvement. In future studies also, the EQ dimensions can be analyzed separately. We also intend to establish the MIC values for EQ-5D in foot and ankle patients to better relate the clinically important improvements.

### BMI and Smoking Habits

It is well established that a high BMI is associated with worse clinical outcomes for hip and knee arthroplasty in terms of pain, disability, and complications.^29^ However, a large retrospective review of 633 patients concluded that the number of complications after forefoot surgery in obese patients was comparable to that in nonobese patients.^37^ The role of body weight in HV outcomes is unclear. In the present study, overweight and obese patients had in general lower SEFAS scores, but the improvement after surgery was similar to normal-weight patients. This is in line with a recently published study showing that confounders such as BMI did not affect surgery outcomes.^16^ However, differences were found in a study by Milczarek et al,^27^ in that the normal-weight patients had slightly better scores on the AOFAS Hallux Metatarsophalangeal Index at the 2-year follow-up, although the results regarding pain (VAS) and subjective foot appearance were similar for both groups. There was no significant difference in complication rate between obese patients and those with normal BMI, and the conclusion of the authors was that HV surgery can be recommended for treating moderate to severe HV, regardless of weight.^27^

A systematic review concluded that smokers who undergo knee and hip arthroplasties are at increased risk of numerous complications, including inpatient mortality, persistent opioid consumption, and worse 1-year patient-reported outcomes.^40^ Another systematic review, which intended to analyze the factors that influence QoL after ankle fractures, showed that smokers had greater rates of disability.^22^ Previous studies have found that complications and nonunion and reoperation rates for hindfoot and midfoot fusions are increased in smokers.^18^ Furthermore, deep infections and wound complications are more frequent. A retrospective study of a variety of forefoot surgeries found that smokers had increased complications, including delayed unions and nonunions.^2^ Krannitz et al^20^ found increased healing time for Chevron osteotomies in smokers. Our study found that smokers had significantly lower SEFAS scores and less improvement at 1- and 2-year follow-ups compared with nonsmokers and those who had quit smoking before surgery. To our knowledge, this has not been demonstrated previously, and further research is warranted. Even though the number of smokers was low (2.4%), active smoking is a modifiable factor and should be discontinued before foot and ankle surgery whenever possible. In Sweden, most foot and ankle surgeons require smoking cessation before hindfoot and ankle surgery. However, the requirements are not as strict for forefoot surgery. Our results reinforce the importance of smoking cessation even in the context of forefoot surgery, including HV, and suggest that Swedish recommendations should be updated.

### Strengths and Limitations

This study has several methodologic strengths and limitations. The major strength of the study is the multicenter register design, with data from a national register and comprehensive data for both 1- and 2-year follow-ups. To our knowledge, the present study is the largest study including 2-year follow-up data. Over the past decades, registries have been used as tools to enhance the quality of medical care. The registries can identify regional variations, provide a realistic view of clinical practice and follow different types of treatments over time. A register-based study includes many surgeons and surgical centers, which means that differences in surgery results may be due to surgeons’ differing approaches and skills (years of experience, number of surgeries performed per year). Additionally, the participants differed from one another in terms of grade of HV, pain and function, and comorbidities. Register-based studies reflect everyday clinical reality and are important complements to other studies with more homogenous cohorts with less variations in terms of surgeons and surgical techniques.

PROMs have received increasing attention in national registries providing a more in-depth personal understanding of the patients perspective regarding the pain, function, HrQoL, and impact of treatment.^33^ Another strength for the study is the use of both generic and a region-specific PROM together with separate specific questions to evaluate patient-reported results. Several authors have pointed out the importance of using PROMs with good measurement properties and to abandon the use of the AOFAS scale.^3,19^ The foot and ankle–specific SEFAS has good measurement properties, and both MIC values and population-based normative values are established for increased usability.^10,12^ From other studies, it is known that the appearance of the foot and possibilities to wear shoes are important matters for patients with HV.^14^ The possibility to use these questions separately is another strength, making it possible to measure satisfaction in several ways.

The most important limitation of this study is the large number of patients with incomplete data from all 3 time points (presurgery and 1 and 2 years after), which resulted in only 18% of eligible patients being fully analyzed. This low response rate may introduce selection bias and limit the generalizability of findings. This limitation reflects the problem that cohort studies have better response rates of PROMs than the register-based ones. Implementation and integration of PROM collection in registers are challenging. From other studies, we know that the highest response rate is found at baseline and decreases as the follow-up time period increases, but also that electronically collected PROMs give a lower response rate than using telephone or paper. Using reminders is also important to increase the response rate.^21,39^

The International Society of Arthroplasty Registries (ISAR) Patient-Reported Outcome measures (PROMs) Working group propose a 60% threshold for an acceptable frequence of response,^32^ but Pronk et al^30^ has in that study discussed whether it is worth the costs required to increase the response rate. There is also no consensus regarding the percentage response rate required to achieve generalizability. At the moment, Swefoot registry has electronically collected PROMs and 2 reminders. Simplifying questionnaires, interacting with patients, and explaining the importance of participation can in the future be other ways to improve the compliance and response rate.

A potential limitation is that 23 participants (3% of the total) had surgery on right and left feet. However, the median time between surgeries was 4 months, and the participants answered surveys for each foot specifically. This subgroup of patients needs to be further evaluated regarding background variables and surgery effects.

## Conclusion

This population-based study with data from Swefoot registry demonstrates that patients surgically treated for HV experienced statistically and clinically significant improvements in SEFAS and EQ-5D scores at both 1 and 2 years postoperatively. Approximately 80% of responding patients expressed satisfaction regarding surgery at both the 1- and 2-year follow-ups. The improvement in SEFAS did not vary based on the severity of HV. Additionally, patients with overweight or obesity showed similar improvements, whereas smokers had significantly lower SEFAS scores and smaller improvements, suggesting the potential benefit of preoperative smoking cessation counseling. Future studies are warranted to explore the factors that affects complications, recurrences, and less favorable results.

## Supplemental Material

sj-pdf-1-fao-10.1177_24730114251351634 – Supplemental material for Patient-Reported Outcomes Before and After Hallux Valgus Surgery: 2-Year Results From a National Registry Study Supplemental material, sj-pdf-1-fao-10.1177_24730114251351634 for Patient-Reported Outcomes Before and After Hallux Valgus Surgery: 2-Year Results From a National Registry Study by Eva Tengman, Cyrus Brodén, Ann-Charlott Söderpalm and Maria C. Cöster in Foot & Ankle Orthopaedics

## References

[1] Abhishek A Roddy E Zhang W Doherty M. Are hallux valgus and big toe pain associated with impaired quality of life? A cross-sectional study. Osteoarthritis Cartilage. 2010;18(7):923-926. doi:10.1016/j.joca.2010.03.011 20417286

[2] Bettin CC Gower K McCormick K , et al. Cigarette smoking increases complication rate in forefoot surgery. Foot Ankle Int. 2015;36(5):488-493. doi:10.1177/1071100714565785 25583954

[3] Bohm ER Kirby S Trepman E , et al. Collection and reporting of patient-reported outcome measures in arthroplasty registries: multinational survey and recommendations. Clin Orthop Relat Res. 2021;479(10):2151-2166. doi:10.1097/CORR.0000000000001852 34288899 PMC8445553

[4] Brooks R. EuroQol: the current state of play. Health Policy 1996;37(1):53-72. doi:10.1016/0168-8510(96)00822-6 10158943

[5] Cai Y Song Y He M , et al. Global prevalence and incidence of hallux valgus: a systematic review and meta-analysis. J Foot Ankle Res. 2023;16(1):63. doi:10.1186/s13047-023-00661-9 37726760 PMC10510234

[6] Cöster M Karlsson MK Nilsson JA Carlsson A. Validity, reliability, and responsiveness of a self-reported foot and ankle score (SEFAS). Acta Orthop. 2012;83(2):197-203. doi:10.3109/17453674.2012.657579 22313352 PMC3339537

[7] Cöster MC Bremander A Nilsdotter A. Patient-reported outcome for 17,648 patients in 5 different Swedish orthopaedic quality registers before and 1 year after surgery: an observational study. Acta Orthop. 2023;94:1-7. doi:10.2340/17453674.2023.6577 36701121 PMC9880767

[8] Cöster MC Bremander A Rosengren BE Magnusson H Carlsson A Karlsson MK. Validity, reliability, and responsiveness of the Self-reported Foot and Ankle Score (SEFAS) in forefoot, hindfoot, and ankle disorders. Acta Orthop. 2014;85(2):187-194. doi:10.3109/17453674.2014.889979 24564747 PMC3967263

[9] Cöster MC Cöster A Svensson F Callréus M Montgomery F. Swefoot - the Swedish national quality register for foot and ankle surgery. Foot Ankle Surg. 2022;28(8):1404-1410. doi:10.1016/j.fas.2022.07.010 35933290

[10] Cöster MC Nilsdotter A Brudin L Bremander A. Minimally important change, measurement error, and responsiveness for the Self-Reported Foot and Ankle Score. Acta Orthop. 2017;88(3):300-304. doi:10.1080/17453674.2017.1293445 28464751 PMC5434599

[11] Cöster MC Rosengren BE Bremander A Brudin L Karlsson MK. Comparison of the Self-reported Foot and Ankle Score (SEFAS) and the American Orthopedic Foot and Ankle Society Score (AOFAS). Foot Ankle Int. 2014;35(10):1031-1036. doi:10.1177/1071100714543647 25015390

[12] Cöster MC Rosengren BE Karlsson MK Carlsson A. Age- and gender-specific normative values for the Self-Reported Foot and Ankle Score (SEFAS). Foot Ankle Int. 2018;39(11):1328-1334. doi:10.1177/1071100718788499 30035614

[13] Coughlin MJ Jones CP. Hallux valgus: demographics, etiology, and radiographic assessment. Foot Ankle Int. 2007;28(7):759-777. doi:10.3113/FAI.2007.0759 17666168

[14] Dawson J Boller I Doll H , et al. Factors associated with patient satisfaction with foot and ankle surgery in a large prospective study. Foot (Edinb). 2012;22(3):211-218. doi:10.1016/j.foot.2012.05.002 22681897

[15] Ferrari J Higgins JP Prior TD. Interventions for treating hallux valgus (abductovalgus) and bunions. Cochrane Database Syst Rev. 2004;1:CD000964. doi:10.1002/14651858.CD000964.pub2 14973960

[16] Hernandez-Castillejo LE Martinez-Vizcaino V Alvarez-Bueno C Quijada-Rodriguez JL Alonso-Galan M Garrido-Miguel M. Effectiveness of hallux valgus surgery on improving health-related quality of life: a follow up study. Foot Ankle Surg. 2022;28(4):431-437. doi:10.1016/j.fas.2021.08.002 34454834

[17] Hernandez-Castillejo LE Martinez Vizcaino V Garrido-Miguel M Cavero-Redondo I Pozuelo-Carrascosa DP Alvarez-Bueno C. Effectiveness of hallux valgus surgery on patient quality of life: a systematic review and meta-analysis. Acta Orthop. 2020;91(4):450-456. doi:10.1080/17453674.2020.1764193 32408787 PMC8023907

[18] Heyes G Weigelt L Molloy A Mason L. The influence of smoking on foot and ankle surgery: a review of the literature. Foot (Edinb). 2021;46:101735. doi:10.1016/j.foot.2020.101735 33168350

[19] Hunt KJ Hurwit D. Use of patient-reported outcome measures in foot and ankle research. J Bone Joint Surg Am. 2013;95(16):e118(1)-e118(9). doi:10.2106/JBJS.L.01476 23965711

[20] Krannitz KW Fong HW Fallat LM Kish J. The effect of cigarette smoking on radiographic bone healing after elective foot surgery. J Foot Ankle Surg. 2009;48(5):525-527. doi:10.1053/j.jfas.2009.04.008 19700113

[21] Levens B Kim BS Aksu N , et al. Young or old age and non-White race are associated with poor patient-reported outcome measure response compliance after orthopaedic surgery. Arthrosc Sports Med Rehabil. 2023;5(6):100817. doi:10.1016/j.asmr.2023.100817 38023444 PMC10661514

[22] Lorente A Pelaz L Palacios P , et al. Predictive factors of functional outcomes and quality of life in patients with ankle fractures: a systematic review. J Clin Med. 2024;13(5):1188. doi:10.3390/jcm13051188 38592026 PMC10932135

[23] Loveday DT Barr LV Loizou CL Barton G Smith G. A comparative prospective cohort health economic analysis comparing ankle fusion, isolated great toe fusion and hallux valgus surgery. Foot Ankle Surg. 2018;24(1):54-59. doi:10.1016/j.fas.2016.11.008 29413775

[24] Menz HB Lord SR. Gait instability in older people with hallux valgus. Foot Ankle Int. 2005;26(6):483-489. doi:10.1177/107110070502600610 15960916

[bibr25-24730114251351634] Menz HB Marshall M Thomas MJ Rathod-Mistry T Peat GM Roddy E. Incidence and progression of hallux valgus: a prospective cohort study. Arthritis Care Res (Hoboken). 2023;75(1):166-173. doi:10.1002/acr.24754 34268894

[26] Mickle KJ Munro BJ Lord SR Menz HB Steele JR. ISB Clinical Biomechanics Award 2009: toe weakness and deformity increase the risk of falls in older people. Clin Biomech (Bristol, Avon). 2009;24(10):787-791. doi:10.1016/j.clinbiomech.2009.08.011 19751956

[27] Milczarek MA Milczarek JJ Tomasik B Laganowski P Nowak K Domzalski M. Being overweight has limited effect on SCARF osteotomy outcome for hallux valgus correction. Int Orthop. 2017;41(4):765-772. doi:10.1007/s00264-017-3419-0 28210803

[28] Nilsdotter AK Cöster ME Bremander A Cöster MC. Patient-reported outcome after hallux valgus surgery - a two year follow up. Foot Ankle Surg. 2019;25(4):478-481. doi:10.1016/j.fas.2018.02.015 30321964

[29] Pozzobon D Ferreira PH Blyth FM Machado GC Ferreira ML. Can obesity and physical activity predict outcomes of elective knee or hip surgery due to osteoarthritis? A meta-analysis of cohort studies. BMJ Open. 2018;8(2):e017689. doi:10.1136/bmjopen-2017-017689 PMC5855486 29487072

[30] Pronk Y Pilot P Brinkman JM van Heerwaarden RJ van der Weegen W. Response rate and costs for automated patient-reported outcomes collection alone compared to combined automated and manual collection. J Patient Rep Outcomes. 2019;3(1):31. doi:10.1186/s41687-019-0121-6 31155689 PMC6545294

[31] Ray JJ Friedmann AJ Hanselman AE , et al. Hallux valgus. Foot Ankle Orthop. 2019;4(2):2473011419838500. doi:10.1177/2473011419838500 PMC8696753 35097321

[32] Rolfson O Bohm E Franklin P , et al. Patient-reported outcome measures in arthroplasty registries. Report of the Patient-Reported Outcome Measures Working Group of the International Society of Arthroplasty Registries Part II. Recommendations for selection, administration, and analysis. Acta Orthop. 2016;87 suppl 1(suppl 1):9-23. doi:10.1080/17453674.2016.1181816 27228230 PMC4937770

[33] Ruseckaite R Mudunna C Caruso M Ahern S. Response rates in clinical quality registries and databases that collect patient reported outcome measures: a scoping review. Health Qual Life Outcomes. 2023;21(1):71. doi:10.1186/s12955-023-02155-5 37434146 PMC10337187

[34] Schrier JC Palmen LN Verheyen CC Jansen J Koeter S. Patient-reported outcome measures in hallux valgus surgery. A review of literature. Foot Ankle Surg. 2015;21(1):11-15. doi:10.1016/j.fas.2014.11.004 25682400

[35] Soderpalm AC Montgomery F Helander KN Cöster MC. Hallux valgus; An observational study on patient characteristics, surgical treatment and pre-operative HRQoL from the Swedish foot and ankle register (Swefoot). Foot (Edinb). 2023;57:102060. doi:10.1016/j.foot.2023.102060 37922633

[36] Spindler FT Ettinger S Arbab D Committee DAFS Baumbach SF. Patient-reported outcome measures in studies on hallux valgus surgery: what should be assessed. Arch Orthop Trauma Surg. 2024;144(11):4745-4752. doi:10.1007/s00402-024-05523-y 39249134 PMC11582207

[37] Stewart MS Bettin CC Ramsey MT , et al. Effect of obesity on outcomes of forefoot surgery. Foot Ankle Int. 2016;37(5):483-487. doi:10.1177/1071100715624209 26747294

[38] Sullivan PW Slejko JF Sculpher MJ Ghushchyan V. Catalogue of EQ-5D scores for the United Kingdom. Med Decis Making. 2011;31(6):800-804. doi:10.1177/0272989X11401031 21422468

[39] Wang K Eftang CN Jakobsen RB Aroen A. Review of response rates over time in registry-based studies using patient-reported outcome measures. BMJ Open. 2020;10(8):e030808. doi:10.1136/bmjopen-2019-030808 PMC7412618 32764078

[40] Yue C Cui G Ma M , et al. Associations between smoking and clinical outcomes after total hip and knee arthroplasty: a systematic review and meta-analysis. Front Surg. 2022;9:970537. doi:10.3389/fsurg.2022.970537 36406352 PMC9666709

[41] Zhu M Chen JY Yeo NEM Koo K Rikhraj IS. Health-related quality-of-life improvement after hallux valgus corrective surgery. Foot Ankle Surg. 2021;27(5):539-542. doi:10.1016/j.fas.2020.07.001 32694077

